# Transcranial Direct Current Stimulation over the Orbitofrontal Cortex Enhances Self-Reported Confidence but Reduces Metacognitive Sensitivity in a Perceptual Decision-Making Task

**DOI:** 10.3390/biomedicines13071522

**Published:** 2025-06-21

**Authors:** Daniele Saccenti, Andrea Stefano Moro, Gianmarco Salvetti, Sandra Sassaroli, Antonio Malgaroli, Jacopo Lamanna, Mattia Ferro

**Affiliations:** 1 Department of Psychology, Sigmund Freud University, 20143 Milan, Italy; saccenti.phd@milano-sfu.it (D.S.); a.moro@milano-sfu.it (A.S.M.); 62103068@mail.sfu.ac.at (G.S.); s.sassaroli@milano-sfu.it (S.S.); 2 Center for Behavioral Neuroscience and Communication (BNC), Vita-Salute San Raffaele University, 20132 Milan, Italy; malgaroli.antonio@unisr.it; 3 Studi Cognitivi, Cognitive Psychotherapy School and Research Center, 20143 Milan, Italy; 4 Clinical Center Tourette Syndrome, IRCCS Ospedale San Raffaele, 20132 Milan, Italy; 5 Faculty of Psychology, Vita-Salute San Raffaele University, 20132 Milan, Italy

**Keywords:** accuracy, confidence, metacognitive sensitivity, decision-making, orbitofrontal cortex (OFC), transcranial direct current stimulation (tDCS)

## Abstract

**Background**: Metacognition refers to the ability to reflect on and regulate cognitive processes. Despite advances in neuroimaging and lesion studies, its neural correlates, as well as their interplay with other cognitive domains, remain poorly understood. The orbitofrontal cortex (OFC) is proposed as a potential substrate for metacognitive processing due to its contribution to evaluating and integrating reward-related information, decision-making, and self-monitoring. **Methods**: This study examined OFC involvement in metacognition using transcranial direct current stimulation (tDCS) while participants performed a two-alternative forced choice task with confidence ratings to assess their metacognitive sensitivity. Before stimulation, the subjects completed the Metacognitions Questionnaire-30 and a monetary intertemporal choice task for the quantification of delay discounting. **Results**: Linear mixed-effects models showed that anodal tDCS over the left OFC reduced participants’ metacognitive sensitivity compared to sham stimulation, leaving perceptual decision-making accuracy unaffected. Moreover, real stimulation increased self-reported confidence ratings compared to the sham. Significant correlations were found between metacognitive sensitivity and negative beliefs about thinking. **Conclusions**: These results highlight the potential involvement of the OFC in the processing of retrospective second-order judgments about decision-making performance. Additionally, they support the notion that OFC overstimulation contributes to metacognitive dysfunctions detected in clinical conditions, such as difficulties in assessing the reliability of one’s thoughts and decision outcomes.

## 1. Introduction

Confidence does not always match ability—some feel secure in a process, even when their performance is no different from others. Indeed, metacognitive knowledge can be defined as the awareness individuals have with respect to their own cognitive processes and their capacity to monitor and reflect on them [1,2]. What we know about our thoughts and how we react to them are pivotal components of psychological functioning, to such an extent that they constitute a watershed between mental health and human psychopathology. Consistent with this speculation, the current literature highlights that attentional bias, impaired self-monitoring, and dysfunctional metacognitive beliefs characterize a variety of psychiatric conditions, including major depression, anxiety disorders, obsessive compulsive disorder, eating disorders, and substance addiction [3,4]. Alongside advancements in the clinical framework, cognitive neuroscience has begun to probe the neural substrates of metacognition, developing ad hoc experimental paradigms, e.g., judgments of learning, feelings of knowing, and confidence judgments (see Fleur et al. [5] for a review), in which the first-order performance is typically followed by a self-reported confidence rating. These paradigms allow for the assessment of individuals’ metacognitive sensitivity, i.e., the extent to which their confidence judgments discriminate between correct and incorrect responses [6,7]. Stated otherwise, metacognitive sensitivity reflects the degree of insight people have into the accuracy of their first-order decision-making or memory performance [8]. On a psychometric level, metacognitive sensitivity is based on the signal detection theory [9], and can be assessed through both parametric and nonparametric indexes (see [10] for a review). Furthermore, this higher-order function is linked to heterogeneous psychiatric symptoms: anxiety and depression are associated with lower confidence and heightened metacognitive sensitivity, whereas compulsive behavior and intrusive thoughts are associated with higher confidence and lower metacognitive sensitivity [11,12].

Lesion studies were among the first to identify the prefrontal cortex (PFC), particularly the frontal lobe, as crucial for second-order judgments about memory performance [13,14]. These findings have been corroborated by subsequent neuroimaging studies, showing a broader frontoparietal network engaged in metacognitive tasks across heterogenous domains, including memory monitoring and perceptual decision-making. Namely, judgments about memory retrieval (e.g., confidence ratings on the successful recognition of previously encoded names or faces) tend to activate the parietal and midline prefrontal regions [15,16], whereas judgments about decision-making accuracy (e.g., confidence ratings regarding the correct categorization of a noisy image as either a face or a house) are associated with the activation of the anterior cingulate cortex (ACC), insula, and lateral anterior PFC [17,18,19]. Structural neuroimaging studies have further contributed to such an exploration, highlighting that visual metacognitive sensitivity correlates with the gray matter volume of the frontal polar regions, whereas changes in memory-related metacognitive sensitivity correlate with the volume of the precuneus [20,21]. Although most of the neuroscientific literature evaluates metacognitive knowledge across dissimilar domains, a body of experimental research has sought to identify the neural substrates of second-order judgments, characterizing and manipulating their temporal focus. Neuroimaging data suggest that the rostral and dorsal regions of the lateral PFC are activated for retrospective judgments (e.g., confidence ratings regarding the correctness of a prior decision about the orientation of a Gabor grating), whereas the medial PFC exhibits greater activation during prospective judgments (e.g., confidence ratings on the probability of successfully recognizing in the future the word that completes a previously encoded sentence) [22,23]. Consistent with these findings, neuromodulation and lesion studies demonstrate that dorsolateral PFC (dlPFC) stimulation and ventromedial PFC damages produce significant alterations in both judgment types [24,25,26,27,28]. However, contradictory results have been obtained even across comparable experimental settings. Indeed, in a previous systematic review, we underscored that prospective and retrospective metacognitive monitoring share certain brain regions, with both activating the anterior and lateral PFC, as well as the ACC and the precuneus [29]. Hence, the neuronal architecture underlying metacognition has yet to be fully elucidated.

A valid approach to fill this gap is to stimulate specific cerebral regions while subjects are engaged in metacognitive tasks. Non-invasive brain stimulation (NIBS) represents in this regard a precious tool for such investigations, since it makes it possible to test the hypothesis that second-order judgments, and thus metacognition, are dependent upon the activity of a specific brain area by transiently modulating its activity without the use of pharmacological agents and evaluating metacognitive effects. Concerning their mechanism of action, NIBS techniques employ electrical and/or magnetic energy to modulate the excitability of the underlying cerebral cortex in a non-invasive fashion and potentially induce long-lasting neuroplastic changes [30]. In NIBS, transcranial direct current stimulation (tDCS) constitutes a promising tool characterized by the application of a low-intensity direct current via scalp-mounted electrodes that modulates neuronal firing rates in a polarity-dependent manner [31]. A suitable target for such intervention in the context of metacognition is the orbitofrontal cortex (OFC), a portion of the ventromedial PFC that remains underexplored in neuromodulation studies due to its limited accessibility and potential discomfort linked with the usage of traditional neurostimulation techniques (e.g., transcranial magnetic stimulation, TMS). Indeed, the OFC processes information about rewards, outcomes, and self-monitoring [32,33,34,35], all of which are likely involved in the making of metacognitive judgments. In addition, the OFC and ACC are essential for the processing of errors and mismatches between expectations and outcomes [36,37], which are both implied in self-assessment and regulation in cognitive tasks. The role of the OFC in processing the emotional aspects of decision-making also supports its potential relevance in metacognitive judgments [38,39], especially in emotionally charged scenarios [40]. Accordingly, previous research highlights a significant correlation between metacognitive accuracy and specific hot executive functions, such as delay discounting [41], i.e., the prioritization of immediate over delayed rewards [42,43]. In this framework, hot executive function refers to affective or reward-related processes, in contrast to cold executive functions, which can be considered as purely cognitive processes, as the latter do not involve prominent emotional arousal and are relatively mechanistic or logically based (e.g., planning and working memory) [44]. The role of the OFC in metacognition, as well as its interplay with hot and cold executive functions, remains, however, poorly understood.

Therefore, the aims of this work were the following: (1) to test the effects of OFC stimulation on metacognitive sensitivity and perceptual decision-making accuracy; and (2) to further investigate how metacognitive sensitivity interacts with the broader aspects of metacognitive beliefs and delay discounting.

## 2. Materials and Methods

### 2.1. Participants

A total of 20 subjects, encompassing 10 males and 10 females, with a mean age of 21.60 ± 1.31 years, were involved in this study. Recruitment by convenience sampling was performed on a word-of-mouth basis at a university site. Most of the participants (*n* = 13) held a high school diploma, while the remaining ones possessed either a bachelor’s degree (*n* = 5) or a master’s degree (*n* = 2). Subjects were eligible for inclusion if they met the following criteria: (1) age greater than 18, (2) being right-handed, (3) with normal or corrected-to-normal vision, and (4) free of medication. The exclusion criteria were as follows: (1) presence of any metallic or electronic implants in the brain/skull (e.g., splinters, fragments, clips, cochlear implants), (2) presence of any metallic or electronic devices at other sites in the body (e.g., cardiac pacemaker, defibrillators, or traumatic metallic residual fragments), (3) previous surgical procedures involving the brain or spinal cord, (4) previous trauma-related brain injury followed by an impairment of consciousness, (5) previous episodes of fainting spells or syncope, (6) previous diagnosis of dermatologic disorders (e.g., dermatitis, psoriasis, or eczema), (7) previous episodes of epileptic seizures, (8) previous diagnosis of other neurological disorders, and (9) current pregnancy or lactation [45]. This study was conducted in accordance with the ethical principles of the Declaration of Helsinki and was approved by the Ethics Board of the Faculty of Psychotherapy Science and the Faculty of Psychology at Sigmund Freud University (protocol number: KCTEQ5BJBSDP4A90246). All subjects were naïve to the aim of the study and provided written informed consent.

### 2.2. Transcranial Direct Current Stimulation

**Figure 1:**
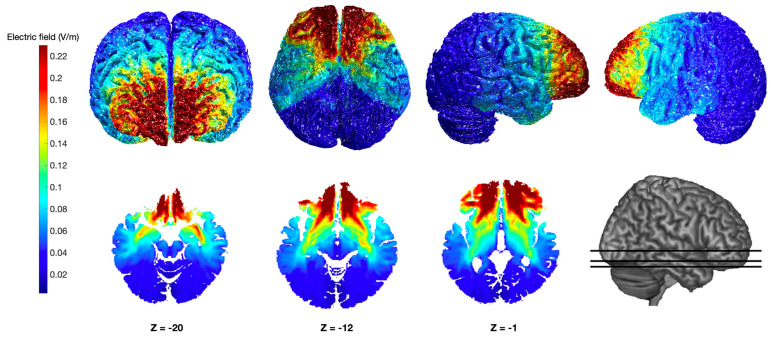
Simulation of the electrical field distribution generated by the transcranial direct current stimulation over the orbitofrontal cortex. Colormap images illustrate the estimated distribution of the electric field, obtained using the anodal Fp1–cathodal Fp2 configuration, at both the cortical and subcortical levels from different viewpoints based on a computational simulation performed with ROAST [52]. The simulation was conducted using the “MNI152_T1_1 mm” template and assuming a current intensity of 2 mA delivered through two 5 × 5 cm^2^ square pad electrodes.

The tDCS current stimulus was delivered using a battery-driven BrainSTIM device (E.M.S. s.r.l., Bologna, Italy) through a pair of 5 × 5 cm^2^ electrodes inserted in separate sponge holding bags and embedded in a saline-soaked solution. The current was applied at a 2 mA constant intensity according to the safety parameters proposed for healthy individuals [46]. The current density (0.08 mA/cm^2^) was also maintained below the safety limits for the entire duration of the stimulation [47]. To stimulate the OFC, the anodal electrode was placed over Fp1, whereas the reference electrode (i.e., cathode) was positioned over Fp2 [48], according to the 10-20 EEG system (see Ly et al. [49]; Yu et al. [50]; Moro et al. [51] for previous studies using this montage). One active stimulation polarity (i.e., anodal Fp1–cathodal Fp2; see Figure 1) and a sham (i.e., no stimulation) condition were adopted.

The active stimulation condition involved continuous current delivery for 20 min, including two 15 s ramping periods at the beginning and end of the stimulation. In contrast, the sham condition did not include actual stimulation, except for two brief ramping stimuli: one at the start and one at the end of the 20 min session. Each of these ramping stimuli consisted of a 15 s increase in current from 0 to 2 mA, followed by a 45 s plateau, and then a 15 s decrease back to 0 mA, mimicking the sensations of stimulation without delivering a sustained current. Indeed, such a procedure has been shown to diminish sensory differences between active and sham stimulation [53].

### 2.3. Psychological Measures

Each participant underwent testing using the Italian version of the short form of the Metacognitions Questionnaire-30 (MCQ-30) [54], which showed satisfactory internal consistency (Cronbach’s α values ranging from 0.71 to 0.87) and convergent validity, as well as good test–retest reliability. The MCQ-30 was used to assess a range of metacognitive beliefs and processes relevant to the vulnerability and maintenance of psychological disorders. The items were rated on a 4-point Likert scale from 1 (‘do not agree’) to 4 (‘completely agree’). Elevated scores were indicative of a greater endorsement of dysfunctional metacognitive beliefs or tendencies, whereas diminished scores reflected fewer dysfunctional metacognitive beliefs or proclivities [55]. The items were grouped into five domains, including cognitive confidence (CC), i.e., confidence in one’s own attention and memory processes; cognitive self-consciousness (CSC), i.e., the tendency to monitor one’s own thoughts and focus attention inward; positive beliefs about worry (POS), i.e., the extent to which an individual thinks that perseverative thinking is useful; negative beliefs about worry concerning uncontrollability and danger (NEG), i.e., the extent to which an individual thinks that perseverative thinking is uncontrollable and dangerous; and beliefs about the need to control thoughts (NC), i.e., the extent to which an individual believes that certain types of thoughts need to be suppressed.

**Figure 2:**
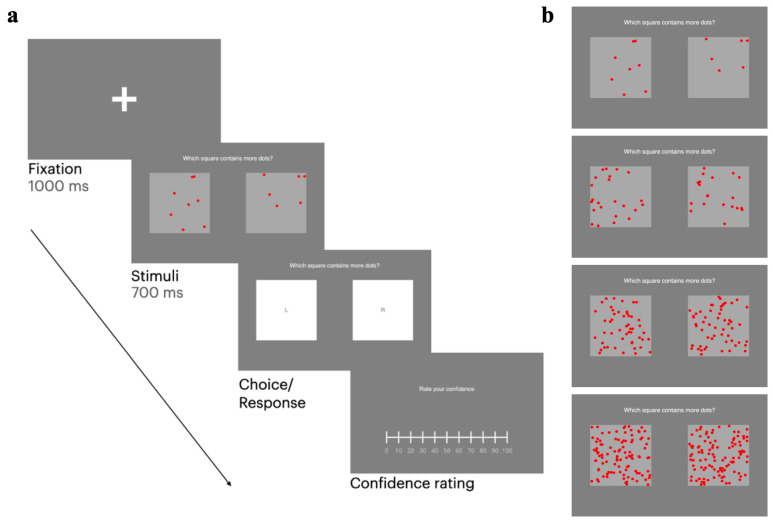
Two-alternative forced choice task with confidence judgments. Subjects were asked to judge which box contained the higher number of dots and indicate their choice by clicking either the “L” or “R” square. Thereafter, they were required to provide a confidence rating for each decision by adjusting the slider (**a**). Four levels of task difficulty were established by ensuring that one randomly selected box contained either 6, 20, 50, or 100 dots, while the other box contained a greater quantity, with a maximum difference of 5 dots (**b**).

Metacognitive sensitivity was evaluated by using a custom-made two-alternative forced choice (2-AFC) task with the addition of confidence judgments developed in PsychoPy (Version 2023.2.3). The task was akin to the one used by Rouault et al. [11] and consisted of two gray squares, each containing a number of salient red dots displayed for 700 ms, following a fixation cross presented for 1000 ms. Participants were asked to judge which of the two boxes contained the higher number of dots and then to report their confidence in each judgment on a granular rating scale ranging from 0 (‘minimum confidence’) to 100 (‘maximum confidence’) (Figure 2). It was explained to the participants that 0 confidence signified a guess response. There was no time limit for either first-order decisions or confidence judgments, and the subjects were not asked to respond as quickly as possible. The participants performed 240 trials, divided into four blocks of 60 trials each. To ensure a progressive increase in task difficulty, each block was further subdivided into four sub-blocks, each consisting of 15 trials. In the first sub-block, one randomly selected box always contained 6 dots, whereas the other contained a variable number of dots, ranging from 7 to 11. In the second sub-blocks, one randomly selected box always contained 20 dots, whereas the other contained a variable number of dots, ranging from 21 to 25. In the third sub-block, one randomly selected box always contained 50 dots, whereas the other contained a variable number of dots, ranging from 51 to 55. In the fourth sub-block, one randomly selected box always contained 100 dots, whereas the other contained a variable number of dots, ranging from 101 to 105 (Figure 2). The fixed difference of 1 to 5 dots across sub-blocks was employed to reduce response bias and prevent the usage of strategic guessing or heuristic-based decision-making strategies. No staircase or calibration procedure to maintain a constant level of performance both during the experiment and across participants was used. On average, the 2-AFC task plus confidence ratings required ~16 min.

Delay discounting was assessed through a custom-made monetary intertemporal choice task (MICT) developed in jsPsych, designed to determine the subjective value, or indifference point (IP), across six temporal delays (i.e., 1, 6, 12, 24, 60, and 120 months) associated with two maximal reward values (i.e., 500 EUR and 10,000 EUR). The adjusted immediate amount procedure described by Holt et al. [56] was used. In each trial, participants were asked to choose between an immediate but adjusted reward and a maximal but delayed one. Based on their choice, the immediate reward was adjusted as follows: if the delayed reward was chosen, the immediate amount increased by half the difference between its current value and the maximum reward; if the immediate reward was chosen, it decreased by the same proportion. This iterative adjustment was repeated over six rounds, after which the IP for the given reward and delay was estimated. The reward and delay were presented randomly. On average, the MICT required ~8 min.

### 2.4. Experimental Design

**Figure 3:**
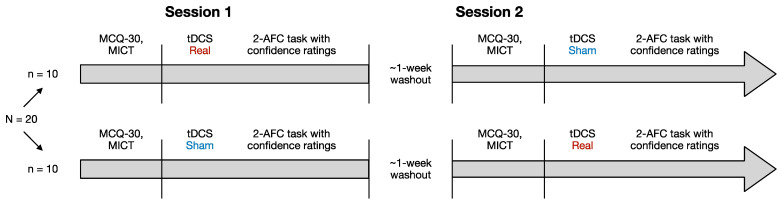
Experimental Procedure. Each subject took part in two experimental sessions. At the beginning of each session, participants completed the Metacognitions Questionnaire-30 (MCQ-30) and a custom-made intertemporal choice task (MICT). Subsequently, half of the participants received the real (active) tDCS protocol, while the other half received the sham tDCS protocol. During the stimulation phase, all participants performed a custom-made two-alternative forced choice (2-AFC) task with confidence ratings. After a 1-week washout period, they returned to the laboratory and completed the 2-AFC task with confidence ratings again, but this time, they received the stimulation protocol opposite to the one administered in their first session.

A double-blind within-subject design was adopted for this study, wherein participants underwent two sessions spaced at least 1 week apart. During the first session, participants completed the MCQ-30 and MICT in a random order. Thereafter, they underwent the first tDCS, which was randomly assigned as either active or sham. Three minutes after the start of stimulation, participants were tested with a custom-made 2-AFC task with the addition of confidence judgments. During the second session, participants fulfilled the MCQ-30 and MICT prior to tDCS and were then subjected to the other stimulation protocol (active or sham) while completing the 2-AFC task plus confidence judgments. The order of active and sham stimulation was counterbalanced across the participants, so that half of them began with the sham condition and the remaining half with the active condition. None of the participants reported sensory differences between the two sessions. See Figure 3 for a graphical representation of the entire experimental procedure.

### 2.5. Statistical Analysis

To conduct the metacognition analysis, two distinct measures of metacognitive sensitivity were used. First, a phi (ϕ) coefficient, i.e., a standard Pearson *r* correlation between accuracy and confidence over the trials of the 2-AFC task [57], was computed for each subject under both conditions. Despite its widespread application [27,58,59,60,61], the ϕ coefficient remains susceptible to contamination by metacognitive bias. In particular, for subjects with a high or low tendency to provide high confidence ratings overall, their ϕ coefficient would likely be altered [10]. Therefore, we also opted for an alternative approach that models the connection between subjects’ performance and metacognition to remove the influence of bias. The meta-*d*′ measure [6,7] was estimated for each subject under both conditions due to its robustness to changes in bias and its propriety to recover simulated changes in metacognitive sensitivity [62]. Such an index has also been employed in previous studies on metacognition [17,21,60,63,64]. Perceptual decision-making accuracy was calculated as the percentage of correct answers given in the 2-AFC task.

To conduct delay discounting analysis, each IP extracted from the MICT was considered. A hyperbolic function, IP = A/(1 + k·Delay) [65], was fitted to each value by means of a nonlinear least-mean squares method. In this model, A corresponds to the reward magnitude (i.e., 10,000 EUR or 500 EUR, depending on the subtask), and Delay represents the temporal intervals of 1, 6, 12, 24, 60, and 120 months. The devaluation coefficient k (expressed in 1/days) was estimated through curve fitting. Conversely, the areas under the curve (AuC) were computed and normalized by expressing the delay as a proportion of the maximum delay and the subjective value as a proportion of the nominal amount, i.e., the subjective value divided by the actual delayed amount [66].

Multiple linear mixed-effect (LME) models were fitted to the data, including metacognitive sensitivity and delay discounting indexes as dependent variables as well as different fixed and random effect factors, as detailed in the Section 3. The LME models were followed by analysis of variance (ANOVA) to extract the exact F- and *p*-values linked to each factor. Kenward–Roger tests were used to compare the fitted LME models. The normality of the ϕ, meta-*d*′, AuC, and k distributions, as well as of the LME model residuals, was evaluated graphically using qqplots and histograms. Due to the skewness of the k distributions, we log-transformed coefficient k, as previously performed in other works [56]. All measures obtained from the tests in all the stimulation conditions were evaluated for the presence of significant correlation using Spearman’s coefficients (ρ). No *p*-value correction method for multiple correlations was applied. All analyses were carried out using custom algorithms developed in R (Version 4.4.0; https://www.r-project.org/ accessed on 1 May 2025).

## 3. Results

### 3.1. Stimulation Effects on Perceptual Decision-Making Accuracy and Self-Reported Confidence

Two different LME models were fitted to these data according to the following formulas (Wilkinson notation):

Y ~ 1 + Protocol × Difficulty + MCQ_30_tot + AUC_Low + AUC_High + (1|Protocol_Order) + (1|subject)(1)

Y ~ 1 + Protocol × Difficulty + (1|Protocol_Order) + (1|subject)(2)

where *y*, the response variable, was set as either the accuracy or confidence in the 2-AFC task. Among the fixed-effect factors evaluated, Protocol indicated whether stimulation was real or the sham, Difficulty indicated the level of complexity within the 2-AFC task, i.e., if the trials comprised 6, 25, 50, or 100 dots in one of the two squares (interaction terms were also included for these two factors), and MCQ_30_tot, AUC_Low, and AUC_High indicated the performances in the related tests. Finally, a random effect of the subject and a random effect of the session order were included.

A comparison of the two models using the Kenward–Roger test showed no significant difference for either accuracy (χ^2^_3_ = 0.7656, *p* = 0.8577) or confidence (χ^2^_3_ = 1.3589, *p* = 0.7152), thus excluding a significant contribution of both delay discounting and higher-order beliefs on subject metacognitive sensitivity in our conditions. Based on such a result, we performed analyses on the more parsimonious model of Equation (2).

**Figure 4:**
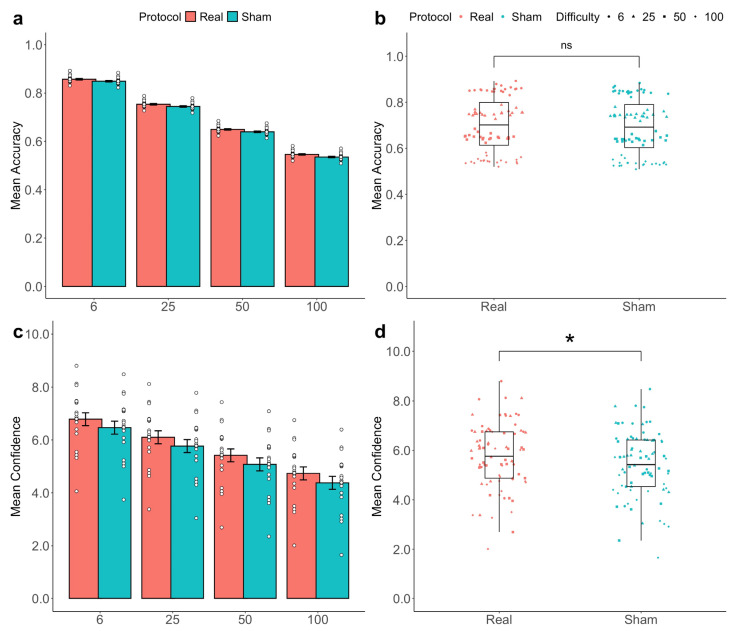
Stimulation effects on accuracy and confidence ratings. A linear mixed-effect model was applied to the data to estimate participants’ metacognitive accuracy and their confidence scores. A barplot of the fitted data depicts the mean accuracy ± SEM in each of the four difficulty levels of the task, revealing a clear trend towards a reduction in the subjects’ perceptual decision-making accuracy (**a**). A boxplot of the fitted data displays the estimated marginal means of the accuracy scores in the two conditions, showing no significant difference in subjects’ perceptual decision-making accuracy between real and sham stimulation (**b**). A barplot of the fitted data displays the mean confidence ± SEM in each of the four difficulty levels of the task, unveiling a clear trend towards a reduction in subjects’ self-reported confidence ratings (**c**). A boxplot of the fitted data shows the estimated marginal means of the confidence ratings in the two conditions, highlighting a significant increase in subjects’ self-reported confidence ratings under real stimulation compared to the sham (**d**). * *p* < 0.05.

ANOVA applied to the fitted models revealed a significant main effect of tDCS and task difficulty level on 2-AFC task confidence (tDCS: *F*(1,154) = 4.9627, *p* = 0.0259; Difficulty: *F*(3,154) = 103.3395, *p* < 0.0001; see Figure 4). Importantly, the main effect of tDCS was not statistically significant when accuracy was set as the response variable (tDCS: *F*(1,154) = 0.0755, *p* = 0.7835; Difficulty: *F*(3,154) = 216.5289, *p* < 0.0001; see Figure 4). Interestingly, no significant interaction effect between these two factors (tDCS and task difficulty level) was found on either accuracy (*F*(1,38) = 0.0064, *p* = 0.9360) or for confidence (*F*(1,38) = 0.0075, *p* = 0.9312).

### 3.2. Stimulation Effects on Metacognitive Sensitivity

To deepen our understanding of the interplay among metacognitive sensitivity, metacognitive beliefs, and delay discounting, the subsequent analysis also encompassed the individual subscales of the MCQ-30. Two different LME models were fitted to these data according to the following formulas (Wilkinson notation):

Y ~ 1 + Protocol × Difficulty + MCQ_30_POS + MCQ_30_NEG + MCQ_30_CC + MCQ_30_NC + MCQ_30_CSC + AUC_Low + AUC_High + (1|Protocol_Order) + (1|subject)(3)

Y ~ 1 + Protocol × Difficulty + (1|Protocol_Order) + (1|subject)(4)

where *y*, the response variable, was set as the ϕ coefficient or meta-*d*′. Among the fixed-effect factors evaluated, Protocol indicates whether the stimulation was real or a sham, Difficulty indicates the level of complexity within the 2-AFC task, i.e., if the trials comprised 6, 25, 50, or 100 dots (an interaction term was also included for these two factors), and AUC_Low, AUC_High, MCQ_30_POS, MCQ_30_NEG, MCQ_30_CC, MCQ_30_NC, and MCQ_30_CSC indicate the performances in the related tests. Finally, a random effect of the subject and a random effect of the session order were included.

A comparison of the two models using the Kenward–Roger test showed no significant difference for either the ϕ coefficient (χ^2^_7_ = 8.6556, *p* = 0.2783) or meta-*d*′ (χ^2^_7_ = 11.6400, *p* = 0.1130), thus excluding a significant contribution of either delay discounting or beliefs about thinking on subject metacognitive sensitivity in our conditions. Based on such a result, we performed analyses on the more parsimonious model of Equation (4).

**Figure 5:**
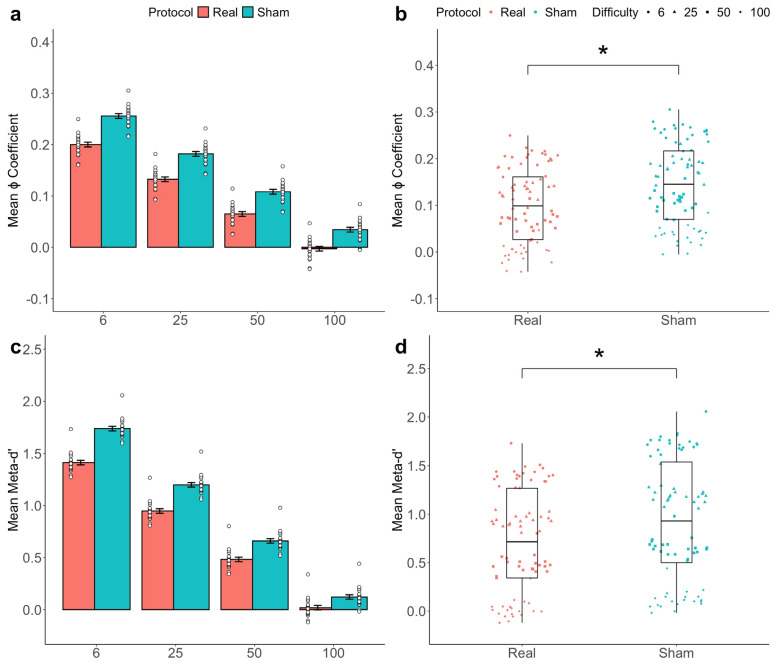
Stimulation effects on metacognitive sensitivity. A linear mixed-effect model was applied to the data to estimate participants’ metacognitive sensitivity scores. A barplot of the fitted data depicts the mean ϕ coefficient ± SEM in each of the four difficulty levels of the task, revealing a clear trend towards a reduction in subjects’ metacognition (**a**). A boxplot of the fitted data shows the estimated marginal means of the ϕ coefficient in the two conditions, indicating a significant reduction in subjects’ metacognitive sensitivity under real stimulation compared to the sham (**b**). A barplot of the fitted data displays the mean meta-*d*′ ± SEM in each of the four difficulty levels of the task, unveiling a clear trend towards a reduction in metacognition (**c**). A boxplot of the fitted data represents the estimated marginal means of meta-*d*′ in the two conditions, highlighting a significant reduction in subjects’ metacognitive sensitivity under real stimulation compared to the sham (**d**). * *p* < 0.05.

ANOVA applied to the fitted models revealed a significant main effect of tDCS and 2-AFC task difficulty level on the ϕ coefficient (tDCS: *F*(1,154) = 5.7218, *p* = 0.0168; Difficulty: *F*(3,154) = 66.3852, *p* < 0.0001; see Figure 5). Importantly, the same results were obtained when meta-*d*′ was set as the response variable (tDCS: *F*(1,154) = 3.9194, *p* = 0.0477; Difficulty: *F*(3,154) = 107.6750, *p* < 0.0001; see Figure 5). Interestingly, no significant interaction effect between these two factors (tDCS and task difficulty level) was found on either the ϕ coefficient (*F*(1,38) = 0.1264, *p* = 0.7223) or meta-*d*′ (*F*(1,38) = 0.5872, *p* = 0.4435).

### 3.3. Summary of the Stimulation Effects on First- and Second-Order Performance

The results obtained from the LME models fitted to the data on subjects’ accuracy, confidence, ϕ coefficient, and meta-*d*′ are summarized in Table 1.

### 3.4. Relationship Between Metacognitive Sensitivity, Metacognitive Beliefs, and Delay Discounting

**Figure 6:**
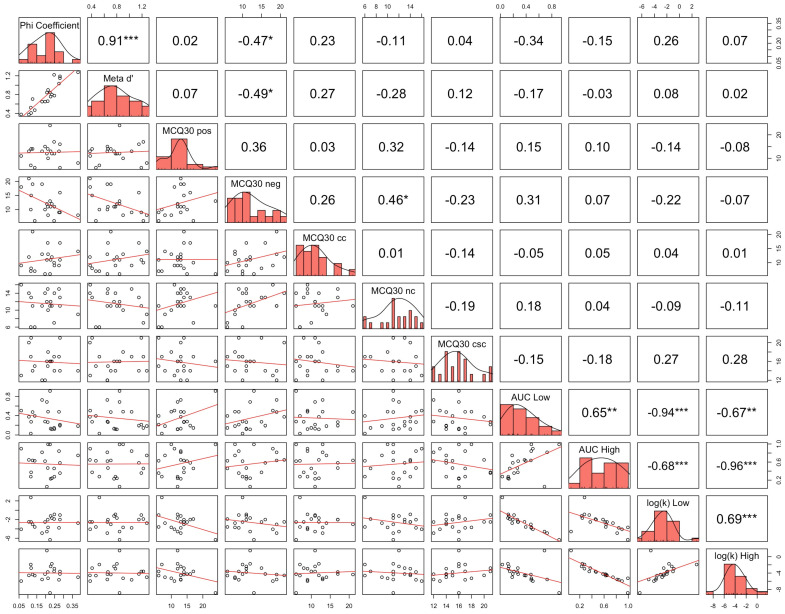
Correlations among metacognitive sensitivity, metacognitive beliefs, and delay discounting under real stimulation. A statistically significant linear relationship was found between the ϕ coefficient and meta-*d*′. Moreover, significant linear relationships were detected among the ϕ coefficient, meta-*d*′, and negative beliefs about thinking. Statistically significant linear relationships were also observed among the AuC and log(k) values linked to low rewards (i.e., 500 EUR) and high rewards (i.e., 10,000 EUR). No statistically significant relationship was found between delay discounting and metacognitive sensitivity, nor between delay discounting and metacognitive beliefs. * *p* < 0.05, ** *p* < 0.01, *** *p* < 0.001.

**Figure 7:**
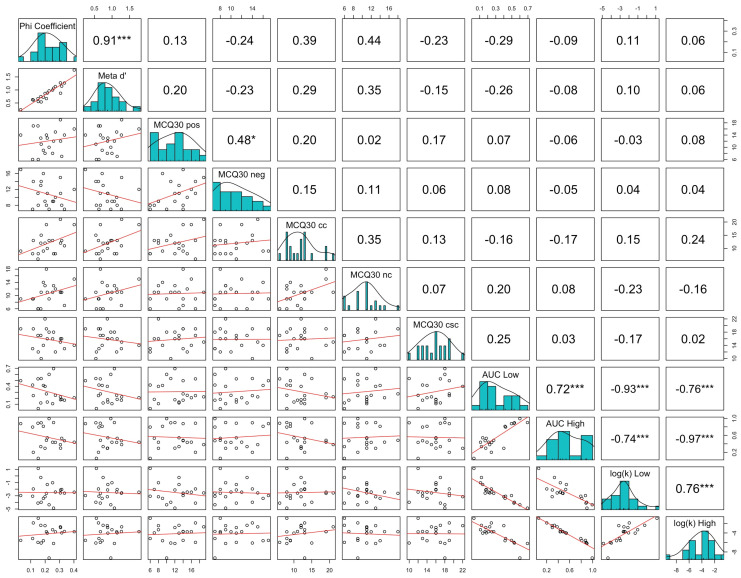
Correlations among metacognitive sensitivity, metacognitive beliefs, and delay discounting under sham stimulation. A statistically significant linear relationship was found between the ϕ coefficient and meta-*d*′. Furthermore, statistically significant linear relationships were observed among the AuC and log(k) values linked to low rewards (i.e., 500 EUR) and high rewards (i.e., 10,000 EUR). No statistically significant relationship was found between delay discounting and metacognitive sensitivity, nor between delay discounting and metacognitive beliefs. * *p* < 0.05, *** *p* < 0.001.

Correlational analysis showed a statistically significant relationship between the ϕ coefficient and meta-*d*′ under both real (ρ = 0.9053, *p* < 0.0001; see Figure 6) and sham stimulation (ρ = 0.9143, *p* < 0.0001; see Figure 7). However, no statistically significant relationship was observed among metacognitive sensitivity and metacognitive beliefs, except for the ones between the ϕ coefficient, meta-*d*′, and negative beliefs about the thinking domain under real stimulation (for ϕ coefficient: ρ = −0.4709, *p* = 0.0361; for meta-*d*′: ρ = −0.4891, *p* = 0.0287; see Figure 6). A statistically significant relationship among the AuC and log(k) values linked to low rewards (i.e., 500 EUR) and high rewards (i.e., 10,000 EUR) was observed under both real (AuC Low and AuC High: ρ = 0.6541, *p* = 0.0023; AuC Low and log(k) High: ρ = −0.6662, *p* = 0.0018; AuC Low and log(k) Low: ρ = −0.9353, *p* < 0.0001; AuC High and log(k) Low: ρ = −0.6812, *p* = 0.0013; AuC High and log(k) High: ρ = −0.9639, *p* < 0.0001; log(k) Low and log(k) High: ρ = 0.6857, *p* = 0.0012; see Figure 6) and sham stimulation (AuC Low and AuC High: ρ = 0.7218, *p* = 0.0005; AuC Low and log(k) High: ρ = −0.7639, *p* = 0.0001; AuC Low and log(k) Low: ρ = −0.9278, *p* < 0.0001; AuC High and log(k) Low: ρ = −0.7398, *p* = 0.0003; AuC High and log(k) High: ρ = −0.9744, *p* < 0.0001; log(k) Low and log(k) High: ρ = 0.7594, *p* = 0.0002; see Figure 7). No statistically significant relationship was found between delay discounting and metacognitive sensitivity, nor between delay discounting and metacognitive beliefs.

## 4. Discussion

The aims of this research were, first, to test the effects of OFC stimulation on metacognitive sensitivity and perceptual decision-making accuracy, and second, to further investigate how metacognitive sensitivity interacts with the broader aspects of metacognitive beliefs and delay discounting. We demonstrated that anodal tDCS application over the left OFC causes a significant reduction in metacognitive sensitivity, leaving perceptual decision-making accuracy unaffected. Such a result highlights the potential involvement of the OFC in metacognition, especially in retrospective second-order judgments about perceptual decision-making performance. Accordingly, in rats, OFC inactivation disrupts metacognitive judgments without affecting decision accuracy [67]. Moreover, TMS studies in humans highlight the involvement of anterior PFC regions in the processing of retrospective second-order judgments without a significant contribution to subjects’ perceptual decision-making performance [28,68]. Considering its established involvement in decision-making [69], the OFC might specifically weight outcomes and process judgments about the value of decisions, thus providing feedback concerning whether an action aligns with one’s own personal goals. Nonetheless, other brain areas, especially the dlPFC, have been implicated in retrospective metacognitive monitoring. Indeed, dlPFC stimulation induces a decrease in subjects’ metacognitive sensitivity, while their perceptual decision-making performance is kept constant [27,63]. Therefore, second-order judgments about decision-making accuracy likely involve a broader network encompassing the OFC, dlPFC, and perhaps other cortical regions. A related hypothesis put forward by an electroencephalography study is that metacognitive decision-making requires an interaction between the frontal and motor areas, painting a picture of metacognition as a process that integrates sensory evidence with information about one’s interactions with the world [70]. Consistent with this evidence, the selective disruption of dorsal premotor cortex activity via TMS reduces individuals’ metacognitive efficiency without any significant alteration in visual discrimination performance [71]. Other candidates for inclusion in this brain network are the intraparietal sulcus and the lateral intraparietal cortex. Notably, boosting intraparietal sulcus/lateral intraparietal cortex-to-V1/V2 back projections using a paired associative TMS protocol improves metacognitive efficiency without affecting motion sensitivity [72]. In our study, we also noted that anodal tDCS over the left OFC induces a significant enhancement in self-reported confidence ratings following first-order decisions. Similarly, single-pulse TMS over the dlPFC produces increased confidence in the absence of relevant changes to accuracy in a visual perceptual decision-making task [64]. This finding suggests that these two brain areas (i.e., the OFC and dlPFC) likely cooperate in processing self-assessments of one’s own decisions, potentially through a mutual interaction with the visual system. Consistently, single-pulse TMS application over the occipital cortex disrupts the internal representation of a visual stimuli but leads to an increase in confidence ratings [73].

To our knowledge, this is the first study that tests the effects of tDCS application over the OFC on retrospective second-order judgments about decision-making accuracy. As a matter of fact, previous tDCS studies mainly focused on the effects of neuromodulation on retrospective memory monitoring. Han et al. [74] made use of high-definition tDCS to seek evidence for a causal role of the ventrolateral PFC in supporting memory advantage for high-value items, finding that the anodal stimulation of this brain region significantly boosts metacognitive sensitivity. Taken together, these pieces of evidence indicate that anodal tDCS application over the ventral portions of the PFC potentially sort opposite effects on second-order judgments about memory retrieval and decision-making accuracy. Transcranial alternating current stimulation (tACS) has also been tested, but its effects on subjects’ retrospective judgments appear more heterogeneous, depending on the task-related domain and the brain areas stimulated during the experiments. Indeed, the application of tACS over the bilateral dlPFC increases both objective cognitive performance and subjects’ self-evaluations in a metamemory task [75], whereas tACS over the occipital cortex produces no effect on metacognition during a visual perception task [76,77]. Further research is, therefore, needed to clarify the differential effects of tDCS and tACS on metacognition.

Our correlational analysis showed a significant relationship between metacognitive sensitivity and negative beliefs about thinking, indicating that under anodal stimulation, individuals that report dysfunctional metacognitive beliefs also struggle to discriminate between correct and incorrect judgments based on their confidence. Namely, when subjects are actively stimulated, their metacognitive beliefs related to the dangerousness and uncontrollability of perseverative thinking correlate more strongly with lower metacognitive sensitivity compared to the control condition. This evidence suggests that an overactivation of the OFC might be linked to clinically relevant alterations in metacognition, including impairments in assessing the reliability of one’s thoughts and the valuation of decision outcomes. Accordingly, subjects with obsessive compulsive disorder consistently report dysfunctional negative beliefs about the uncontrollability and dangerousness of their worry [78], and show hyperactivity of the OFC [79,80], which has been, in turn, associated with exaggerated mental representations of anticipated aversive events [81]. Compulsive behavior and intrusive thoughts have also been associated with higher confidence and lower metacognitive efficiency in a perceptual decision-making task administrated in a large general population sample [11]. Nevertheless, experimental studies investigating second-order judgments about decision-making accuracy in clinical patients are still lacking [12,82], and they could be implemented in the future to enhance the understanding of how these subjects evaluate their own decisions and how this relates to their symptomatology.

Although previous neuromodulation studies have demonstrated the involvement of the OFC in the processing of delay discounting [51,83], our data did not reveal any consistent or significant correlation between metacognitive sensitivity and delay discounting in the two conditions (i.e., anodal and sham stimulation). Hence, these functions could be unrelated, at least when considering retrospective second-order judgments about decision-making performance. An alternative approach could involve continuing to investigate the relationship between the OFC, delay discounting, and prospective metacognitive information prior to decision-making [41,84]. Even though NIBS investigations targeting the OFC are still lacking, there is, in fact, a long tradition in imaging and lesion studies underlining the involvement of the ventromedial PFC in metacognition, especially in prospective second-order judgments about memory performance [26,85,86,87,88,89]. To provide a comprehensive portrait of this phenomenon, future research could, therefore, shift its focus to the understanding of the neural correlates of prospective metacognitive judgments in perceptual decision-making tasks [90].

Our study suffers, however, from significant limitations. First, although the metacognition analysis was performed on more than two hundred trials for each subject, this study included twenty participants, resulting in low statistical power. Second, metacognitive sensitivity was assessed using a custom-made 2-AFC task with the addition of confidence ratings, which could benefit from further testing, particularly by manipulating the four randomly chosen difficulty levels that appeared to play a crucial role in the second-order judgments about decision-making accuracy. Third, a correlational measure was used to assess metacognitive sensitivity in the first stance (i.e., ϕ coefficient), leaving nonparametric measures, such as the area under the type-2 ROC curve, unexplored. Since the latter were shown to be influenced by task performance [9,91], we opted for subsequently modeling the connection between performance and metacognition by employing the meta-*d*′ measure. This metric offers advantages such as ease of interpretation and effective control over the influence of performance on metacognitive sensitivity [7]. However, although meta-*d*′ shares the same units as (type 1) *d*′, the two measures were not directly compared in the present study. Fourth, baseline anxiety and depressive symptom severity were not assessed, even though an association between these clinical dimensions and higher-order cognitive functions, including delay discounting, confidence, and metacognitive sensitivity [11,92], has been reported in the scientific literature. Future investigations could fill this gap by identifying subgroups of respondents to tDCS-based interventions based on their pre-treatment cognitive profile and subclinical symptomatology.

Apart from its limitations, our study incorporates significant clinical implications. Indeed, stimulating the OFC via anodal tDCS could ameliorate the symptomatology of those patients whose confidence or metacognitive sensitivity deviates from the normative range, e.g., subjects with obsessive compulsive disorder [93], depressive disorders [82], or substance addiction [41]. An effective approach to be further tested in clinical populations could involve combining tDCS with psychotherapeutic interventions aimed at modifying metacognitive beliefs and removing attentional bias, such as metacognitive therapy [94]. Multimodal treatments could, in fact, reduce psychiatric symptom severity through a metaplastic route [95]—that is, synaptic changes induced by psychotherapy in neural circuits are likely to facilitate subsequent synaptic modifications elicited by NIBS (and vice versa), thus enhancing both their likelihood and long-term stability. Integrated NIBS–neuroimaging approaches could aid in the definition and localization of such plasticity-related phenomena, thereby shedding light on the physiological effects exerted by tDCS in humans or in animal models of psychiatric and neurological disorders.

## Figures and Tables

**Table 1:** Summary of the LME model results, showing the means ± SEM for each difficulty level and for the pooled data across the real and sham stimulation conditions.

Response Variable	Difficulty Level	Real (*n* = 20)	Sham (*n* = 20)	*F*-Value	*p*-Value
Accuracy	6	0.857 ± 0.003	0.849 ± 0.003	-	-
	25	0.753 ± 0.003	0.744 ± 0.003	-	-
	50	0.650 ± 0.003	0.640 ± 0.003	-	-
	100	0.546 ± 0.003	0.535 ± 0.003	-	-
	Pooled	0.702 ± 0.009	0.692 ± 0.009	0.006	0.936
Confidence	6	6.785 ± 0.245	6.465 ± 0.245	-	-
	25	6.102 ± 0.245	5.771 ± 0.245	-	-
	50	5.420 ± 0.245	5.076 ± 0.245	-	-
	100	4.737 ± 0.245	4.382 ± 0.245	-	-
	Pooled	5.760 ± 0.278	5.420 ± 0.278	4.963	0.026
ϕ coefficient	6	0.200 ± 0.005	0.256 ± 0.005	-	-
	25	0.132 ± 0.005	0.182 ± 0.005	-	-
	50	0.065 ± 0.005	0.108 ± 0.005	-	-
	100	−0.003 ± 0.005	0.034 ± 0.005	-	-
	Pooled	0.099 ± 0.016	0.145 ± 0.016	5.722	0.017
meta-*d*′	6	1.413 ± 0.022	1.738 ± 0.022	-	-
	25	0.948 ± 0.022	1.199 ± 0.022	-	-
	50	0.483 ± 0.022	0.660 ± 0.022	-	-
	100	0.019 ± 0.022	0.122 ± 0.022	-	-
	Pooled	0.716 ± 0.110	0.930 ± 0.110	3.919	0.048

## Data Availability

The raw data supporting the conclusions of this article will be made available by the authors on request.

## Abbreviations

The following abbreviations are used in this manuscript:

PFC	Prefrontal cortex
ACC	Anterior cingulate cortex
dlPFC	Dorsolateral prefrontal cortex
NIBS	Non-invasive brain stimulation
tDCS	Transcranial direct current stimulation
OFC	Orbitofrontal cortex
TMS	Transcranial magnetic stimulation
MCQ-30	Metacognitions Questionnaire-30
CC	Cognitive confidence
CSC	Cognitive self-consciousness
POS	Positive beliefs about worry
NEG	Negative beliefs about worry
NC	Need to control thoughts
2-AFC	Two-alternative forced choice
MICT	Monetary intertemporal choice task
IP	Indifference point
AuC	Area under the curve
LME	Linear mixed-effect (models)
ANOVA	Analysis of variance
ROAST	Realistic volumetric approach to simulate transcranial electric stimulation
SEM	Standard error of the mean
tACS	Transcranial alternating current stimulation
V1/V2	Primary and secondary visual cortex
ROC	Receiver operating characteristic (curve)
